# Cell-type-specific modelling of intracellular calcium signalling: a urothelial cell model

**DOI:** 10.1098/rsif.2013.0487

**Published:** 2013-09-06

**Authors:** Peter A. Appleby, Saqib Shabir, Jennifer Southgate, Dawn Walker

**Affiliations:** 1 Department of Computer Science, Kroto Research Institute, University of Sheffield, Sheffield S3 7HQ, UK; 2 Jack Birch Unit for Molecular Carcinogenesis, Department of Biology, University of York, York YO10 5DD, UK

**Keywords:** epithelial cells, urothelial cells, calcium signalling, P2X, P2Y

## Abstract

Calcium signalling plays a central role in regulating a wide variety of cell processes. A number of calcium signalling models exist in the literature that are capable of reproducing a variety of experimentally observed calcium transients. These models have been used to examine in more detail the mechanisms underlying calcium transients, but very rarely has a model been directly linked to a particular cell type and experimentally verified. It is important to show that this can be achieved within the general theoretical framework adopted by these models. Here, we develop a framework designed specifically for modelling cytosolic calcium transients in urothelial cells. Where possible, we draw upon existing calcium signalling models, integrating descriptions of components known to be important in this cell type from a number of studies in the literature. We then add descriptions of several additional pathways that play a specific role in urothelial cell signalling, including an explicit ionic influx term and an active pumping mechanism that drives the cytosolic calcium concentration to a target equilibrium. The resulting one-pool model of endoplasmic reticulum (ER)-dependent calcium signalling relates the cytosolic, extracellular and ER calcium concentrations and can generate a wide range of calcium transients, including spikes, bursts, oscillations and sustained elevations in the cytosolic calcium concentration. Using single-variate robustness and multivariate sensitivity analyses, we quantify how varying each of the parameters of the model leads to changes in key features of the calcium transient, such as initial peak amplitude and the frequency of bursting or spiking, and in the transitions between bursting- and plateau-dominated modes. We also show that, novel to our urothelial cell model, the ionic and purinergic P2Y pathways make distinct contributions to the calcium transient. We then validate the model using human bladder epithelial cells grown in monolayer cell culture and show that the model robustly captures the key features of the experimental data in a way that is not possible using more generic calcium models from the literature.

## 1. Introduction

Modelling studies have been pivotal to understanding the origin of different calcium patterns and how they are modulated by external stimuli (reviewed by [1,2]). The models described to date may be classified in a number of ways, for example, by the number of intracellular calcium pools they contain, or by the mechanisms used to generate the calcium transient. These models are capable of reproducing a variety of experimentally observed calcium transients, ranging from spikes and bursts to rapid oscillations [3–12] and even chaotic behaviour [13]. Together, these studies have provided important insights into the mechanisms that drive calcium transients and the various types of transient that can be produced.

In non-excitable cells, calcium signalling depends upon an integrated system of receptor activation, ion channel opening, influx of exogenous calcium into the cell, release from internal stores and restoration of ionic equilibria. The pathways involved are generally well preserved. For example, a key mechanism involved in the production of calcium transients in non-excitable cells is the release of calcium from the endoplasmic reticulum (ER). Typically, a small elevation in the cytosolic calcium concentration triggers release of calcium via a number of pathways, for example, ryanodine- and IP_3_-receptor-activated calcium channels. This leads to a rapid and much larger elevation in cytosolic calcium concentration. The sharp rise in calcium concentration subsequently inhibits the calcium release mechanism which, in conjunction with pumping of calcium into the ER and into the extracellular medium, leads to a rapid falling phase. The overall pattern is that of a sharp calcium spike. Of particular interest is the oscillatory behaviour recorded in the cytosolic calcium concentration in response to external application of a receptor-binding agonist; such behaviour has been observed in a wide variety of non-excitable cell types [14].

In addition to its fundamental role in regulating basic cell processes, calcium signalling also plays a major role in coordinating tissue homeostasis and in effecting many specialized cell-type-specific differentiated functions. This raises the question as to how a single ion is able to mediate such a diverse range of cellular responses. Identifying the key components of the calcium signalling apparatus in a particular cell type and understanding how they interact to contribute to the calcium transients generated is of central importance to understanding these higher function processes. In our case, we are interested in the self-repairing barrier epithelium of the urinary bladder and, in particular, understanding how the precise temporal and spatial profile of calcium elevation in the tissue in response to wounding influences the subsequent coordinated behaviour of proliferation and migration at the cellular level. Although existing calcium signalling models contain many of the components we require, no single model integrates all of the pathways known to be important in this cell type. A model from the literature will therefore be unlikely to capture existing experimental data relating to urothelial cells. More importantly, it will not be capable of generating predictive results for this cell type. Both qualities are of central importance if our aim is to explain how the precise form of a calcium transient can direct the subsequent behavioural response of a cell.

Here, we use the urothelial cell as an exemplar for developing a cell-type-specific model of ER-dependent calcium signalling. The model relates the cytosolic, extracellular and ER calcium concentrations via a system of ordinary differential equations. The resting cytosolic calcium concentration is a function of the leak current from the ER, and pumping terms that transfer calcium from the cytosol to the ER and the extracellular medium. When stimulated by an external agonist, calcium influx occurs owing to opening of ligand-gated calcium channels in the cell membrane and owing to calcium release from the ER via the IP_3_R pathway. The following pathways are therefore integral to our model:

— Leak current: this represents the leak of calcium into the cytosol from the ER.
— SERCA pumping: this represents the active removal of calcium from the cytosol and transference to the ER via SERCA pumps.
— Calcium pumping: this represents the pumping of calcium out of the cytosol into the extracellular medium through various pumps and exchangers in the cell membrane, such as the plasma membrane Ca^2+^ ATPase and sodium calcium exchanger. In urothelial cells, this is an active process that drives the cytosolic calcium concentration to a target value [15].
— Ionic pathway: this represents the influx of calcium due to ion channel activation.
— P2Y–IP_3_R pathway: this represents the activation of P2Y receptors by extracellular ATP, and the translation of this activity into IP_3_ production. The IP_3_ subsequently triggers the release of calcium from the ER into the cytosol via IP_3_R channel activation.

**Figure 1.**
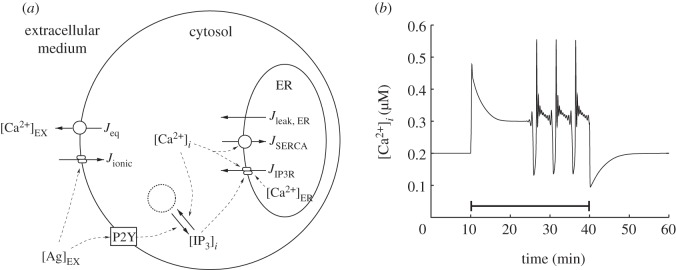
The cell model and an example calcium transient. (*a*) Diagram of the whole cell calcium signalling model. The model describes the evolution of extracellular, cytosolic and ER calcium concentrations. Leak currents between these compartments are represented by straight arrows, pumped currents by arrows overlayed with circles, and channel currents by arrows overlayed with two ellipses. The square represents the P2Y receptors that are activated by the external agonist and drive IP_3_ production from an inexhaustible pool of intracellular resources, indicated by the dotted circle. Dashed lines indicate couplings between the various components of the model. (*b*) An example calcium transient produced by the model with an extracellular calcium concentration of 2 mM using the parameter set in the electronic supplementary material, table S1. Agonist (25 μM) is applied at *t* = 10 min and removed at *t* = 40 min, as indicated by the solid line.

Some of these components have already been examined in the modelling literature. Wherever possible, we draw upon these studies, integrating descriptions of components that are known to be present in our cell type and known to be conserved in their function. Examples are the SERCA pump and the IP_3_R pathway which have been the focus of several previous studies [5,12,13,16]. In addition, we integrate descriptions of several additional pathways that play a specific role in urothelial cell signalling. These additional terms are formulated specifically to capture the calcium dynamics of urothelial cells. Examples are the description of ATP-activated P2X channels in the cell membrane, representing the pathway allowing calcium influx owing to ion channel activation [17], and the active calcium pumping process that drives the cytosolic calcium concentration to equilibrium [15]. A sketch of the model is shown in figure 1*a* and, the full mathematical details of its implementation are given in the electronic supplementary material.

In this paper, we focus on quantifying the ability of this model to produce calcium transients and examining how their nature changes in response to variation of the model's parameters. In the Results section, we show that our model preserves much of the dynamics of the more generic models upon which it is based and is capable of generating spikes, bursts, oscillations and sustained elevations in the cytosolic calcium concentration. However, the precise nature of the dynamics, including the transitions between qualitatively distinct response modes, is unique due to the specific combination of pathways we model and due to the presence of urothelial-cell-specific terms. We explore how the nature of the calcium transients depends on the parameters of the model, with particular emphasis on those we expect to vary significantly between individual urothelial cells. We also show that, novel to our model, the ionic and P2Y pathways make distinct contributions to the calcium transient. Finally, we apply the model to a specific experimental system consisting of human bladder urothelial cells grown in monolayer cell culture. We validate this form of the model by comparing it with experimentally recorded calcium transients and show that the model robustly captures the key features of the calcium transients observed in low and high extracellular calcium concentrations in a way that is not possible using more generic models from the literature.

## 2. Results

We examine the evolution of the cytosolic calcium concentration in the model in response to application of an external agonist. Default parameters are given in the electronic supplementary material, table S1 and, unless otherwise noted, the extracellular medium has a fixed physiological calcium concentration of [Ca^2+^]_EX_ = 2 mM. Combined with a target equilibrium cytosolic calcium concentration of [Ca^2+^]*_i_* = 0.2 μM, this leads to a equilibrium ER calcium concentration of [Ca^2+^]_ER_ = 5.2 μM. These equilibrium calcium concentrations also determine the initial concentrations at the start of each simulation. The initial cytosolic IP_3_ concentration is always set to zero.

### 2.1. Calcium transients produced by the model: plateaus, spikes and bursts

We first examine the overall form of the calcium transients that the model can produce when the cell is stimulated by application of an external agonist. We record the virtual cytosolic calcium concentration over 60 min, with 25 μM of agonist applied at *t* = 10 min and removed at *t* = 40 min. This test protocol provides an extended period of stimulation that uncovers the full dynamical response of the model. Figure 1*b* shows an example of a cytosolic calcium transient triggered by this protocol using the parameter set in the electronic supplementary material, table S1. Prior to the application of the agonist, the cell is at equilibrium. When the agonist is applied at *t* = 10 min, an initial upward fluctuation in the cytosolic calcium concentration occurs. During this fluctuation, the concentration first rises to around 0.5 μM then falls to a plateau at around 0.3 μM. This plateau is unstable, and after a period of time, the cell enters a bursting mode. Following removal of the agonist at *t* = 40 min, we observe a final downward fluctuation in the cytosolic calcium concentration, followed by recovery to baseline. The form of this calcium transient is similar to that observed in other models of calcium signalling [3–6,13]. The particular combination of terms we have used to construct our model, and the addition of urothelial-cell-specific terms, therefore preserves the fundamental dynamics of the models on which our study is based. By altering the parameters of the model, a wide variety of different transients can be produced that contain the same basic components of an initial peak, extended plateau and a subsequent bursting or spiking mode.

### 2.2. Robustness analysis using one-at-a-time variation of parameters

The precise form of the calcium transient produced by our model is strongly dependent upon the choice of parameters. In figure 1*b,* we specifically chose parameters that generate a bursting response similar to that observed in other calcium models in the literature. However, many of the parameters in the electronic supplementary material, table S1 are only very loosely constrained by the experimental data or are derived from modelling studies that focused on a different cell type. We are therefore interested in understanding in detail how the calcium transient changes as a function of the parameters of the model. Our model has a total of 18 parameters. Nine of these parameters are physical constants that can be expected to have the same value for all of the cells in a population, for example, receptor activation rates, dissociation constants and Hill coefficients. The remaining nine parameters describe quantities such as maximum calcium currents, and therefore include dependencies on quantities such as receptor or channel numbers which we expect may vary significantly from cell to cell.

**Figure 2.**
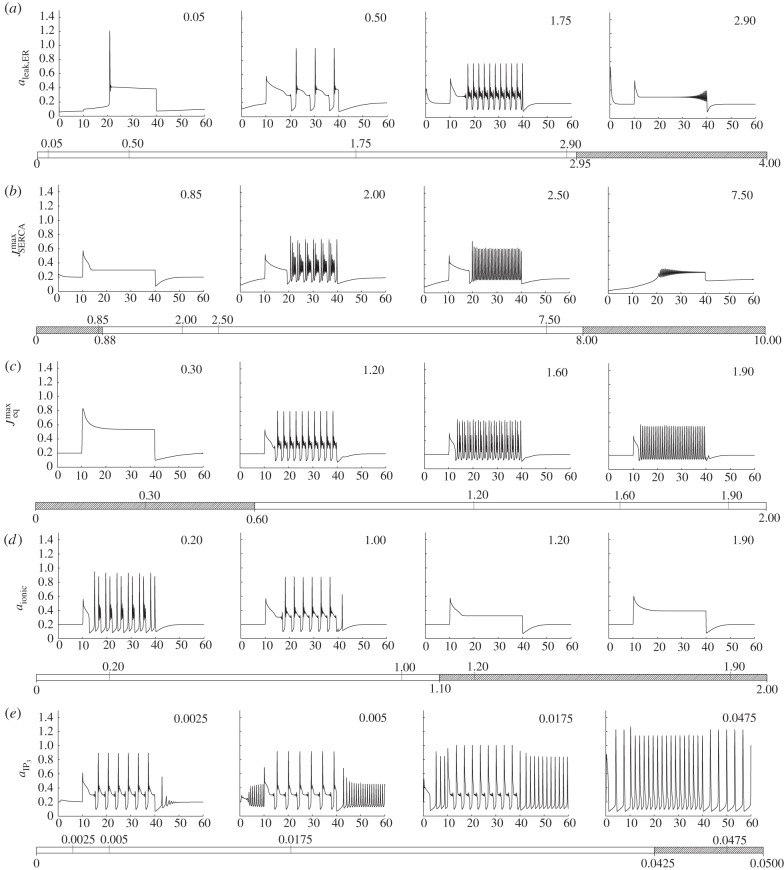
The model contains a total of 18 parameters, nine of which we expect to vary significantly from cell to cell. Here, we show a one-at-a-time robustness analysis of the first five of these parameters. From (*a*–*e*), these are *a*_leak,ER_, 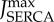, 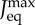, *a*_ionic_ and 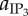. Each plot shows the cytosolic calcium concentration (vertical axis, μM) as a function of time (horizontal axis, minutes), and each row shows how the transient changes as one of these parameters is varied. In each case, four example plots are shown corresponding to the upward ticks on the bar below the plot, which also indicates the range of each parameter expressed in units of the default values given in the electronic supplementary material, table S1 (with the exception of *a*_IP_3__, which uses absolute values). The shaded areas of the bars correspond to parameter regimes that produce qualitatively different calcium transients compared with the baseline case, with the transitions between them indicated by the downward ticks.

**Figure 3.**
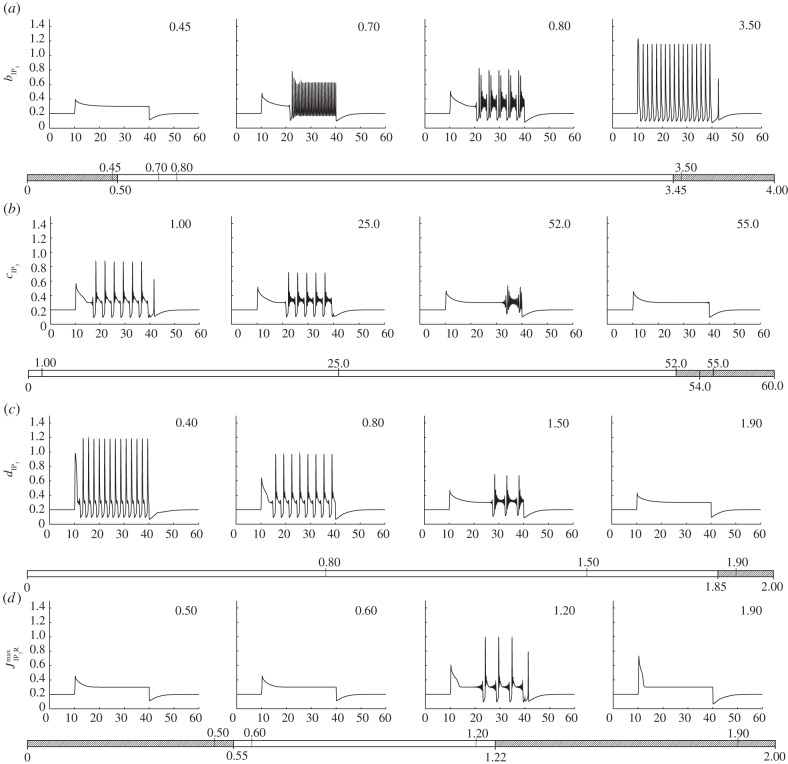
One-at-a-time robustness analysis of the remaining four parameters which we expect to vary significantly from cell to cell. From (*a*–*d*) these are 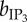, 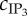, 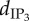 and 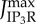.

**Figure 4.**
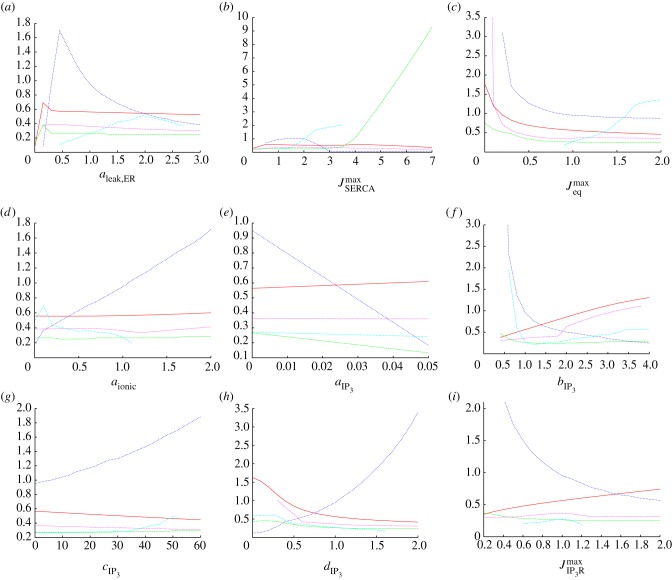
One-at-a-time robustness analysis for the nine parameters (*a*–*i*) examined in figures 2 and 3 using the transient classifier described in the electronic supplementary material. Each plot shows the magnitude (solid line, vertical scale in mM) and time (long dashed line, min) of the initial peak, the time taken for the calcium to fall to 80% of this initial peak (short dashed line, min), the magnitude of the subsequent plateau (dotted line, mM) and the rate of spiking or bursting that follows (dot-dashed line, events per minute). These five quantities capture the key features of the calcium transient. (Online version in colour.)

We first examine the latter nine parameters. To assess the role of these nine parameters, we first vary each of them, in turn, and examine how the qualitative form of the calcium transient changes. The result of this one-at-a-time robustness analysis is shown in figures 2 and 3. Each parameter is bounded at zero but, in all cases, there is no well-defined upper limit. We have therefore chosen a range of values that displays the different dynamics that the model can produce when each parameter is varied, truncating our investigation when no further qualitative changes are observed. Further insights into the role of each parameter can be achieved by extracting key features of the calcium transient and plotting these as a function of each parameter. In the electronic supplementary material, a calcium transient classification scheme is described in which transients are characterized by the magnitude and time of the initial peak, the time taken for the calcium to fall to a specified fraction of this initial peak,^1^ the magnitude of the subsequent plateau and the rate of spiking or bursting that follows. Figure 4 shows the result of applying this analysis to the calcium transients produced by our model, indicating how each of the five key outputs of our model changes as a function of the nine parameters of interest.

By examining figures 2–4 together, the role of each parameter in shaping the resulting calcium transient can be understood. A detailed analysis of how the dynamics of our model are influenced by each of the nine parameters we expect to vary significantly from cell to cell is presented in the electronic supplementary material. Summarizing this analysis, we conclude that a wide range of peak heights, peak widths and transitions between qualitatively distinct modes (e.g. the onset or cessation of spiking) can be produced. The parameters *a*_leak,ER_, and 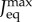 both influence the resting cytosolic calcium concentration but have a qualitatively different impact on the form of the calcium transient produced as they are varied. The same observation applies to *a*_ionic_, 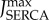 and 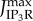, which together determine the dynamic calcium currents that give rise to transients in response to stimulation by an agonist, and 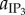, 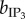, 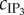 and 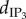, which determine the IP_3_ dynamics. By varying these parameters, the model is capable of producing a wide range of calcium transients with differences in the frequency and amplitude of bursting and spiking, and the transition between bursting- and plateau-dominated modes.

An analogous robustness analysis of the remaining nine parameters in the model is shown in the electronic supplementary material, figures S1–S3. In contrast to the nine parameters described above, these parameters are not expected to vary significantly from cell to cell. The purpose of this analysis is therefore to understand the stability of our model in the region of parameter space around the default parameter values given in the electronic supplementary material, table S1. As an example consider *k*_SERCA_, which is the SERCA dissociation constant. Figures S1 and S3 in the electronic supplementary material show that although some changes in the bursting rate occur the dynamics of the model are qualitatively the same if this parameter is scaled up or down by as much as 30%. Thus, the shape of the transient shown in figure 1*b* is largely preserved for values of *k*_SERCA_ from 70% to 130% of the value given in the electronic supplementary material, table S1. Similar observations apply to 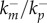, *n*, *k_p_*, *k_d_*, *k_a_* and *k_y_* which can all be scaled in a range of at least 80–145% of the values in the electronic supplementary material, table S1. In many cases, the range of acceptable values is much larger. We conclude that, while the dynamics of the model are affected by changes in these parameters, we would have to choose values that are significantly different to the representative values given in the electronic supplementary material, table S1, which are based either on experimental data or on values typically used in the modelling literature, before the dynamics begins to be strongly affected.

The two exceptions to this trend are ~Ca, the equilibrium cytosolic calcium concentration, and *k_z_*, the dissociation constant for the ER-calcium-dependent scaling factor of the IP_3_R current. ~Ca must not be larger than approximately 110% of the value in the electronic supplementary material, table S1, otherwise bursting is prevented owing to the rapid breakdown of any IP_3_ that is produced. *k_z_*, on the other hand, must not be less than approximately 95% of the value in the electronic supplementary material, table S1 or the IP_3_R current is no longer gated by the ER calcium concentration, which also prevents bursting from occurring. A value of 0.2 μM for ~Ca is similar to the equilibrium cytosolic calcium concentrations used by other modelling studies in the literature [4–6,11–13]. This value has also been experimentally measured in a variety of cell types and found to typically lie in the 0.05–0.2 μM range [18–21]. As the value of 0.2 μM is at the top of end of this experimentally measured range, we expect that the transition that occurs at higher values will not play a significant role in most cell types. *k_z_* is less well constrained. However, the gating dependence of the IP_3_R current on the ER calcium concentration is known to occur at physiological ER calcium concentrations, and the presence of this gating is also required for spiking or bursting in other models in the literature. We therefore proceed using the value of *k_z_* given in the electronic supplementary material, table S1, noting the requirement for this gating to be present when interpreting the results that follow.

### 2.3. Multivariate sensitivity analysis

The one-at-a-time robustness analysis presented above provides insights into how the dynamics of our model depend on individual parameters in the electronic supplementary material, table S1. We are also interested in understanding how the dynamics change when more than one parameter is varied at the same time. Performing a multivariate sensitivity analysis for all 18 parameters at once is not feasible and we must therefore choose a subset to investigate. The nine parameters examined in figures 2–4 are certain to vary significantly within the cell population as they each incorporate a dependence on receptor or channel numbers, and as such they form the natural focus of our attention. Of these nine parameters, *a*_IP_3__, *c*_IP_3__ and *d*_IP_3__ determine IP_3_ degradation and basal production rates, and 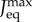 as well as 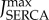 influence the resting cytosolic and ER calcium concentrations. Although these parameters play a central role in determining the equilibrium state of our virtual cell, which, in turn, influences the shape of any subsequent calcium transients, they are not directly influenced by external stimuli, which primarily drives the ionic and P2Y pathways. We therefore focus on the parameters *a*_ionic_, 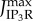 and 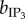. These parameters determine the magnitude of the ionic calcium current that permits calcium flow into the cytosol from the extracellular environment, the magnitude of the IP_3_R calcium current that permits calcium flow into the cytosol from the ER and the rate of IP_3_ production due to P2Y receptor activation, respectively. In other words, together, they determine the relative magnitude of the ionic and P2Y pathways. We vary *a*_ionic_, 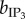 and 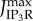 across the same range of scaling values as used in figures 2–4. For each value, we choose values for the remaining two parameters using a Latin hypercube sampling (LHS) approach [22]. LHS reduces the number of simulations that we are required to run while still thoroughly sampling the parameter space, producing a more manageable set of data points.

**Figure 5.**
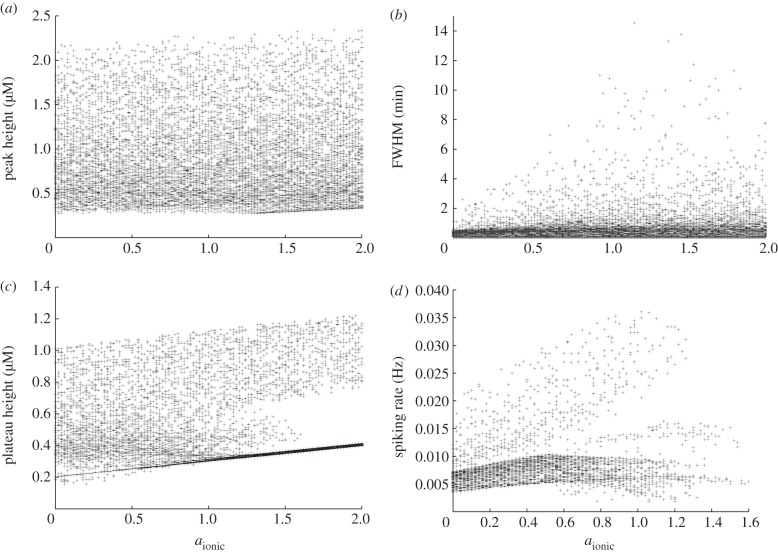
Multivariate sensitivity analysis using *a*_ionic_ as the governing parameter. (*a*) Peak height, (*b*) FWHM (the time taken for the calcium to fall to 80% of the initial peak), (*c*) plateau height and (*d*) spiking rate, if spiking or bursting occurs.

**Figure 6.**
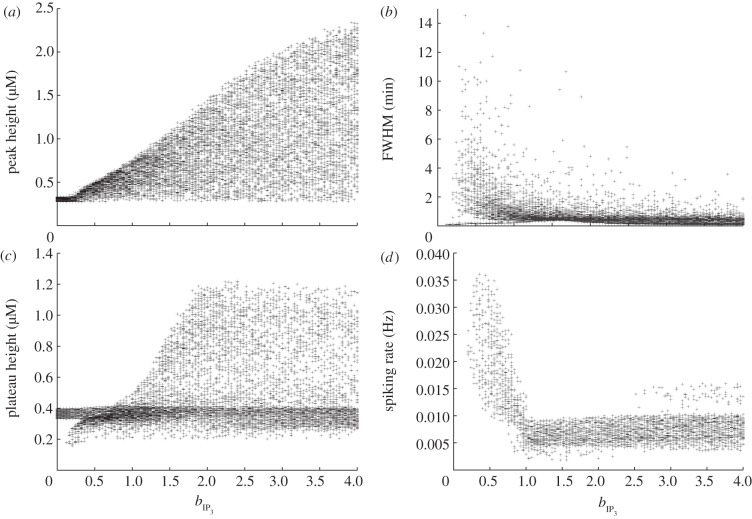
Multivariate sensitivity analysis using *b*_IP_3__, as the governing parameter. (*a*) Peak height, (*b*) FWHM, (*c*) plateau height and (*d*) spiking rate, if spiking or bursting occurs.

**Figure 7.**
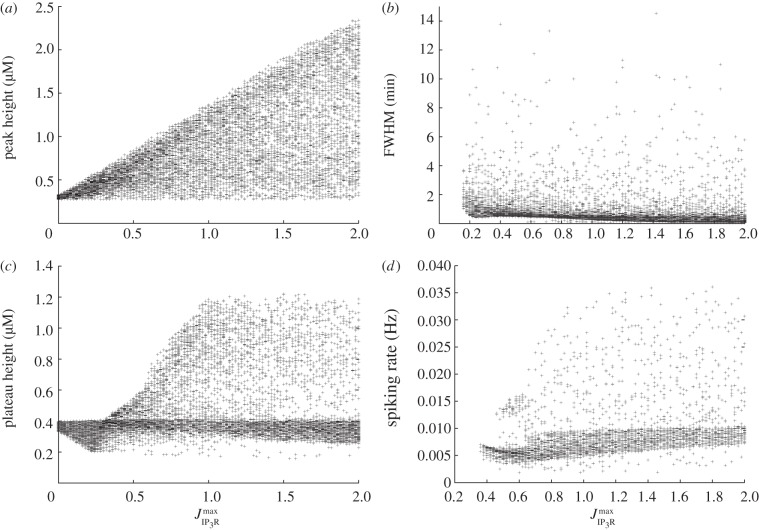
Multivariate sensitivity analysis using 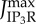 as the governing parameter. (*a*) Peak height, (*b*) FWHM, (*c*) plateau height and (*d*) spiking rate, if spiking or bursting occurs.

Figures 5, 6 and 7 show the result of this sensitivity analysis for *a*_ionic_, 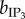 and 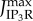, respectively. In each figure, panel (*a*) shows how the initial peak height depends on the governing parameter. In figure 5, we see an almost uniform distribution of points, indicating the initial peak height is not strongly constrained by the value of *a*_ionic_. This supports our conclusion from figure 2 that the initial peak height is largely insensitive to this parameter. The corresponding plots from figures 6 and 7 show that the maximum initial peak height is strongly limited by both 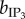 and 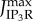 displaying a sigmoidal and linear dependence, respectively. Again, this supports the observation from figure 3 that the initial peak height increases as a function of both parameters. We conclude that, in our model, the initial peak is driven by the P2Y pathway via IP_3_ signalling that triggers release of calcium from the ER, and that the ionic pathway plays very little role in shaping this feature of the transient. Panel (*b*) in figures 5, 6 and 7 shows the dependence of the full-width half-maximum (FWHM) of the initial peak on the three governing parameters. The FWHM indicates how long the initial peak in cytosolic calcium concentration takes to fall to (in our case) 80% of its maximal value. As *a*_ionic_ increases the maximum, FWHM increases but very few data points occupy these higher values. Instead, the majority of data points lie in a band with a small and approximately constant FWHM. This indicates the FWHM, for the majority of parameter sets, is largely unaffected by the ionic pathway. Figure 6 shows a gamma-like distribution of data points, with the value of FWHM strongly limited when 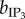 becomes large. Figure 7 shows that the FWHM is largely insensitive to 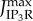, indicating that once the ER has released its calcium stores it does not play an important role in sustaining the subsequent plateau. Taken together, panels (*a*) and (*b*) show that the nature of the initial peak of the transient is largely determined by the parameters 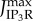 and 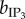. Specifically, the initial peak height increases with 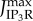, whereas for small values of 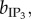 the peak is small and longer-lasting but, for larger values, the peak is larger but briefer. Thus, although both 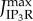 and 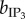 influence the P2Y–IP3 pathway they make distinct contributions to controlling the resulting calcium transient.

Panel (*c*) in each figure illustrates how the plateau height depends on each of the governing parameters. As *a*_ionic_ increases, there is a clear overall trend towards higher plateau values. The plots for both 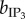 and 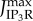 are more complicated, but two broad trends can be observed. First, there is a dense band of points with a plateau height of 0.4 that stretches the full width of the plot. This indicates that for a significant number of parameter combinations, the plateau height is largely unaffected by changes in either 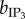 or 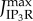. In addition, there is a second, sparser set of points that increases in a broad, sigmoidal-like relationship with the governing parameter. This second branch indicates that modes exist in which a much higher plateau height can be produced, gated by both 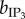 and 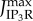. This reveals a much more complicated dependence of the plateau height on these parameters than the one-at-a-time robustness analysis indicated. Comparison of the occupancy of these two branches reveals that the large majority of data points lie in the band with an approximately constant plateau height of around 0.4. The points in the upward sweeping branch correspond to cases where both *a*_ionic_ and 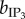 are towards the top end of their specified range. In other words, the model typically resides in the regions of parameter space where the plateau height is independent of 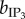 and 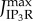. We conclude that for typical parameter sets the overall magnitude of the plateau is largely determined by the ionic pathway with the P2Y pathway playing very little role in shaping this feature of the transient, but we note that remoter regions of parameter space exist where this is not necessarily the case.

Finally, panel (*d*) in each figure illustrates how the spiking or bursting rate depends on each of the three parameters. The bursting or spiking behaviour is due to the cyclic emptying and refilling of calcium from the ER, and we therefore expect the spiking rate to be largely determined by the IP_3_R and SERCA currents. However, examining figure 5 reveals an additional dependence on the ionic pathway. Spiking can occur for all values of *a*_ionic_ we examine, but, as this parameter increases, we see the emergence of two distinct branches of data points. In one branch, there is an overall linear trend towards a higher firing rate. This branch is sparsely occupied, however, and the majority of data points lie in a branch that first increases in firing rate, then decreases once the *a*_ionic_ exceeds around 50% of the default value in the electronic supplementary material, table S1. This indicates that the ionic current does indeed play a role in the spiking mode of our model. The overall sparseness of the data points also increases for large *a*_ionic_ values, indicating that the spiking mode can be prevented if the ionic current is sufficiently large. These observations are consistent with those made from the single parameter robustness analysis shown in figures 2–4 while illustrating that, for a limited number of regions in parameter space, spiking is still possible with a strong ionic current. By contrast, spiking is strongly gated by both 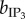 or 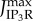 which are both required to be above a minimum value for spiking to occur. As 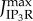 increases, there is a general but slow trend towards a high firing rate. The dependence on 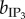 is more complicated, with the onset of rapid spiking followed by a transition to a slower firing rate, which remains approximately constant independent of any further increases in 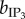.

In summary, the three key parameters in our model, *a*_ionic_, 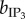 and 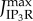, play separate but partially overlapping roles in shaping the calcium transient. The ionic current plays a dominant role in determining the height of the plateau but plays little role in determining the initial peak height or duration. The P2Y pathway plays a dominant role in shaping the initial peak but, in typical regions of parameter space, plays very little role in determining the plateau height. The spiking or bursting mode is strongly influenced by the P2Y pathway, but the ionic pathway also makes a significant contribution to determining the spiking rate and a very strong ionic current can suppress spiking entirely. By changing 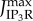, 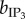 and *a*_ionic_, which we expect to vary significantly from cell to cell, it is possible to move between modes made up of different combinations of these components and modes that are dominated by one component entirely. In §2.4, we show that the interplay of these two pathways plays a central role in shaping experimentally observed calcium transients in cultured urothelial cells.

### 2.4. Validation of the model using normal human urothelial cell cultures

We have established that our urothelial cell model can generate a range of calcium transients and shown how the key parameters in the model influence their shape. We have also shown that the ionic and P2Y pathways make partially separable contributions to the transient. Having developed this general understanding of the model's dynamics, we are interested in applying it to a specific test case. This allows us to verify that the formulation of our model is correct by comparing the results to real experimental data.

**Figure 8.**
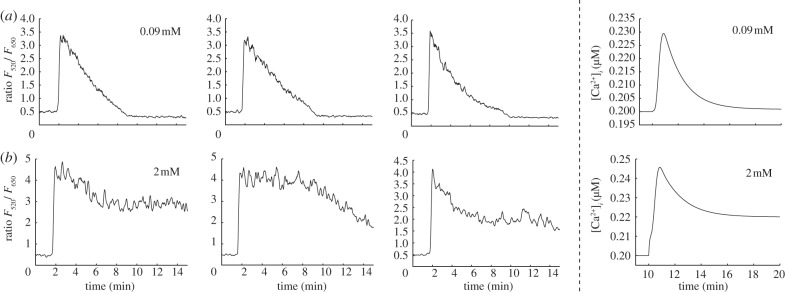
Comparison of calcium transients observed in cultured normal human urothelial cells and generated by the theoretical model in different extracellular calcium concentrations. (*a*) Application of ATP with [Ca^2+^]_EX_ = 0.09 mM triggers transients that are composed of an initial upward fluctuation in cytosolic calcium concentration followed by a rapid return to baseline. The first three panels show examples of experimentally recorded transients. The right most panel shows a transient generated by the model. The transient is very similar in nature to the experimental data, with an initial peak followed by a rapid return to baseline. Parameters have been set to the default values in the electronic supplementary material, table S1, with the exception that *a*_ionic_, 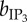 and 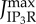 have been scaled by factors of 0.2, 0.4 and 0.2, respectively. (*b*) With [Ca^2+^]_EX_ = 2 mM, the transients that are composed of an initial peak followed by an extended plateau. The first three panels show examples of experimentally recorded transients. The right most panel shows the theoretical result, using the same parameter set that produced the transient in row A. The model successfully captures the transition to a peak-plateau response observed in the experimental data.

Here, we examine the calcium transients evoked in cultured urothelial cells triggered by the application of the purinergic agonist ATP, the principal extracellular signalling factor released during wounding of a urothelial cell monolayer. We first use a stimulation protocol consisting of 100 μM of ATP applied for 15 mins as described in the electronic supplementary material. Figure 8*a* shows three examples of experimentally recorded calcium transients with an extracellular calcium concentration of 0.09 mM. The transients produced in this low-calcium environment are characterized by a large initial peak in the cytosolic calcium concentration followed by a relatively rapid return to the baseline concentration. The cytosolic calcium concentration is not sustained at a greatly elevated level for any length of time and there is an absence of bursting or spiking, features that were observed in the entire sampled population (*n* = 8). An example transient produced by the theoretical model in the same, low calcium environment is also shown. Parameters have been set to the default values in the electronic supplementary material, table S1, with the exception that *a*_ionic_, 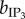 and 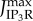 have been scaled by 0.2, 0.4 and 0.2, respectively. The transient produced by the model is qualitatively very similar to the experimental data, with a large initial peak in the cytosolic calcium concentration followed by a relatively rapid return to baseline.

Figure 8*b* shows three examples of experimentally recorded calcium transients using an identical stimulation protocol in the presence of a higher (physiological) extracellular calcium concentration of 2 mM. In this case, we observed a plateau-dominated transient. The key features of this transient are an initial peak followed by an extended plateau which persists as long as the agonist is present. The transient is therefore qualitatively distinct to that produced in the lower extracellular calcium concentration, which lacked the sustained elevation of cytosolic calcium concentration following the initial peak. An example transient produced by the theoretical model in this higher extracellular calcium concentration is also shown. Parameters are the same as used to produce the transients in figure 8*a*. The transient produced by the model is again qualitatively very similar to the experimental data, with a large initial peak followed by an extended plateau.

**Figure 9.**
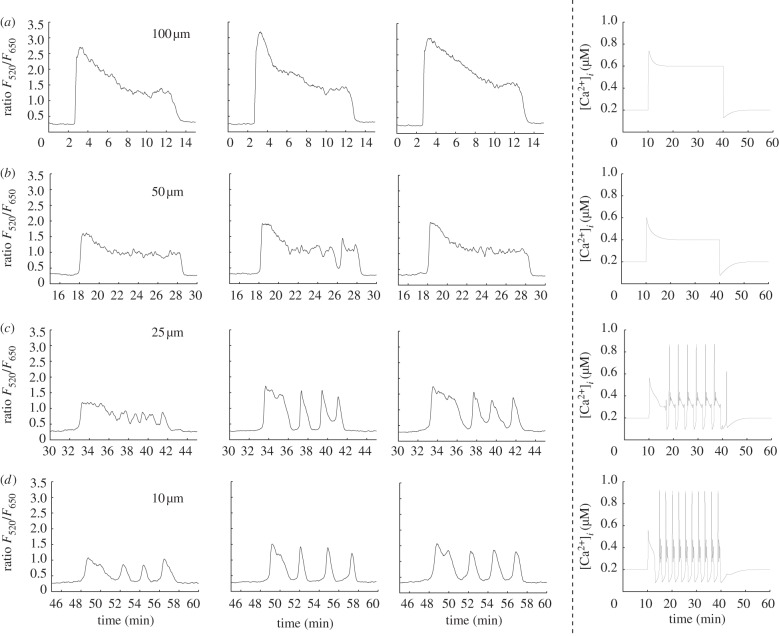
Comparison of calcium transients in cultured normal human urothelial cells and generated by the model using different concentrations of ATP in 2 mM extracellular calcium. (*a*) 100 μM ATP triggers transients that are composed of an initial upward fluctuation in cytosolic calcium concentration followed by an extended plateau. (*b*) Subsequent application of 50 μM ATP produces transients with a smaller initial peak and plateau. In the second cell, the plateau begins to break down after the 25 min mark, indicating the emergence of periodic behaviour. (*c*) ATP (25 μM) triggers an initial transient followed by periodic behaviour, which is most fully developed in cells two and three. (*d*) Application of 10 μM ATP triggers transients with a small initial peak and plateau followed by periodic, spiking behaviour in all three example cells. Right column: transients generated by the model using the same virtual stimulation protocol. Parameters have been set to the default values in the electronic supplementary material, table S1, with the exception that *a*_ionic_, 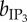 and 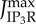 have been scaled by factors of 0.3, 0.4 and 0.5, respectively. The model successfully captures the transition from a large peak-plateau response to a smaller peak-plateau response, followed by a transition into a qualitatively distinct spiking mode.

**Figure 10.**
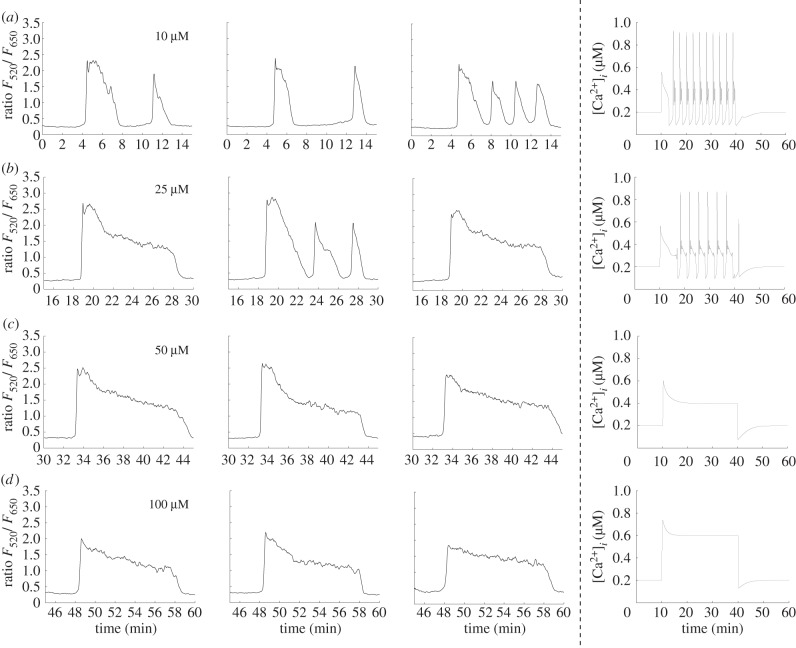
Comparison of calcium transients triggered by the application of different concentrations of ATP in 2 mM extracellular calcium. Reversing the order of application of the different ATP concentrations used in figure 9 generates the opposite behaviour: a qualitative shift from a spiking dominated mode to peak-plateau-dominated mode. The first three columns show examples of experimentally recorded cells, the right most column shows theoretical transients generated by the model. Parameters are the same as in figure 9.

As a second validation of our model, we use a stimulation protocol consisting of four different ATP concentrations applied in succession for 10 min each. This allows us to observe how changing the magnitude of stimulation alters the calcium transient in individual cells. Figure 9 shows three examples of experimentally recorded calcium transients with an extracellular calcium concentration of 2 mM using ATP concentrations of 100, 50, 25 and 10 μM applied in sequence. With 100 μM ATP, the transients are characterized by a large initial peak in the cytosolic calcium concentration followed by an extended plateau. Following a brief recovery period, the subsequent application of 50 μM ATP triggers transients that have a similar overall shape but are smaller in magnitude in both the initial peak and plateau. In the second cell, the plateau begins to break down towards the end of the stimulation period, indicating the onset of periodic behaviour in this particular cell. Subsequent application of 25 μM ATP triggers transients that have an initial fluctuation followed by entry into a periodic mode that, in the first cell, is superimposed over a plateau. Finally, 10 μM ATP triggers an initial fluctuation followed by entry into a periodic mode for all of the cells we observed. As the concentration of ATP is reduced, the overall pattern of behaviour is a shift from a large magnitude peak-plateau response to a smaller magnitude peak-plateau response then entry into a qualitatively distinct spiking mode. The right column of figure 9 shows transients generated by the model using an identical virtual stimulation protocol. Parameters have been set to the default values in the electronic supplementary material, table S1, with the exception that *a*_ionic_, 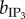 and 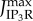 have been scaled by factors of 0.3, 0.4 and 0.5, respectively. The model successfully captures the transitions from a large magnitude peak-plateau response to a smaller magnitude peak-plateau response to a spiking mode observed in the experimental data. Figure 10 shows a similar experimental-theoretical comparison except that the order of application of the different ATP concentrations has been reversed (10, 25, 50, then 100 μM ATP). We see the inverse behaviour: a qualitative shift from a spiking mode at low ATP concentrations to a peak-plateau response as the concentration becomes larger is preserved, indicating that both this experimental observation and the ability of our model to reproduce it are robust.

There is considerable variability in the responses of different cells. Although more exact matches can be achieved in figures 8, 9 and 10 by tuning a larger number of parameters in the model, this would not be particularly useful in terms of verifying the overall ability of the model to capture the distinct types of calcium transients we observe. We have therefore restricted our investigation to scaling the three parameters *a*_ionic_, 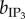 and 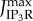, leaving the remaining parameters at the default values given in the electronic supplementary material, table S1. The choice of scaling factors was made empirically by examining various combinations and using the set that gave the best overall match to the data. Despite this restriction, the model is capable of capturing the key features of the experimentally observed calcium transients, specifically the transition from a peak-baseline response to a peak-plateau response when the extracellular calcium concentration is increased and the transition from a spiking to peak-plateau response when the ATP concentration is increased with a fixed extracellular calcium concentration. Simulations show that more generic calcium signalling models taken from the literature are not capable of reproducing these results as they do not contain a description of all of the pathways incorporated in our model [4–6,11–13]. In particular, these models lack the active pumping mechanisms that drive the cytosolic calcium concentration to a target value, and the ionic and P2Y pathways that are critically important for generating and sustaining the plateau mode in our model.

## 3. Discussion

In this paper, we have presented a one-pool model of ER-dependent calcium signalling in urothelial cells. The model relates the cytosolic, extracellular and ER calcium concentrations through mechanistic descriptions of the leak currents, SERCA pumping, calcium pumping, ionic pathway and the P2Y − IP_3_R pathway. Although many calcium signalling models exist in the literature very rarely have cell-type-specific models been constructed and directly linked to the underlying biology. It is important to show that this can be achieved within the theoretical framework adopted by our model, and the models upon which it is based. The model presented here builds on the description of the SERCA, P2Y and IP_3_ pathways developed in the existing models and incorporates an explicit description of ATP-activated P2X channels in the cell membrane, which represents the pathway for calcium influx due to ion channel activation, and an active calcium pumping process that drives the cytosolic calcium concentration to a target equilibrium value. The model is capable of generating a wide variety of calcium transients, the precise nature of which is strongly influenced by the interplay of the ionic and P2Y pathways. Importantly, this urothelial-specific model can reproduce the transitions in experimentally recorded calcium transients from cultured normal human urothelial cells due to changes in the extracellular calcium concentration in a way that is not possible with more general, or more abstract, models from the literature.

The model examined here produces a wide range of responses, including spikes, bursts and sustained elevations in cytosolic calcium concentration. Similar behaviours can be produced by other models in the literature. However, many of these behaviours are preceded by a slow initial transient and only become fully developed over timescales of tens of minutes. In our simulations, we are free to probe the full response of the model and uncover these behaviours, but, experimentally, it is more difficult to stimulate cells for extended periods. Continued application of ATP can lead to exhaustion of intracellular calcium stores, bleaching of the dyes used to visualize the cells or even apoptosis. Stimulation of urothelial cells *in situ* also tends to be briefer in duration lasting, for example, a few minutes during a wounding protocol [23]. In this sense, it is the early stage behaviours such as the initial peak, decay to baseline and sustained plateaus that are likely to be the most physiologically relevant.

As with any modelling study, a number of simplifications have been made during the formulation of our model. For example, there is no downregulation of the ionic or P2Y-receptor pathways in response to extended agonist application. Nor have we included mitochondrial calcium stores, or store-operated channels, in this initial formulation of the model. Although there is some evidence for the presence of mitochondrial calcium stores in urothelial cells, it is unclear how strong a role they play in driving the calcium dynamics. Including mitochondrial or other subcellular stores could subtly shape the calcium signal in urothelium cells under prolonged agonist stimulation and play a role in cell death in the extreme cases of uncontrolled intracellular calcium increases. However, as the precise interplay between the ER and mitochondria is not fully understood in urothelial cells, we chose to formulate our model without them. The model also makes the assumption that quantities such as the cytosolic calcium and IP_3_ concentrations are single-valued. In other words, the model does not include a consideration of the physical structure and geometry of the cell and organelles within it. The spatial properties of the cell and the role that diffusion of cytosolic calcium could, in principle, play an important role in the dynamics of the calcium signal. Both experimental and modelling studies have shown that the calcium puffs and sparks observed in some cells types are spatially dependent, and an accurate description of these phenomena would require a detailed spatial model. However, this is not necessarily the case with our cell type and we must have a strong motivation to include such a level of detail in our model. A simple model has the virtue of being easier to understand and, if correctly formulated, will still capture the behaviours of interest (in our case, the calcium transients) without introducing unnecessary layers of complexity. As we have shown in the Results section, the model presented here is capable of robustly capturing the key features of experimentally recorded calcium transients in cultured normal human urothelial cells, indicating an appropriate level of detail in its formulation.

An advantage of formulating a mechanistic model is the potential to generate predictive results that can be tested experimentally. One approach to generating experimentally verifiable predictions is to make simulated pharmacological manipulations. In this type of simulation, a particular pathway is attenuated or blocked entirely by changing one or more of its governing parameters. As the model contains explicit representations of each of the pathways known to be important in epithelial cell signalling, a particular pathway can be targeted in a way that mimics the action of a particular pharmacological agent used in the laboratory. The simulation results are then directly comparable with the experimental data. As an example, our model contains an explicit representation of the ionic and P2Y pathways, allowing us to probe the model at any point along either of these pathways. We may, for example, attenuate or activate the ionic pathway at the receptor level, or choose to intervene further downstream in the calcium-dependent gating of the IP_3_R channel. Similarly, we can influence the P2Y pathway at the receptor activation level or further downstream at the IP_3_ production stage. If these pathways were described using a single term or were formulated in a more abstract way (as is the case in more generic models in the literature), then this would not be possible. Another example is the SERCA pumping mechanism, which in the model described here can be altered in a way that corresponds closely to the type of manipulations available in the laboratory. Such predictions can be used to further verify the model and to constrain parameter choices, and we intend to pursue experiments of this nature in future work.

Our broader research is focused on understanding the mechanisms governing tissue homeostasis and repair. Using the wounding of normal human urothelial cell cultures as an experimental system, it has been shown that calcium waves are coordinated across the population and that the calcium signature exhibited by an individual cell is dependent on local context and reflective of the emergent cellular response. The type of joint theoretical–experimental study that we have undertaken is essential for understanding the dynamics of calcium signalling in urothelial cells and the complexities of the integrated population response that lies at the heart of tissue homeostasis. Having developed this model, the next step will be to understand how the time profile of cytosolic calcium concentration can direct subsequent cell behaviour. This will require the use of a large-scale, agent-based system to permit exploration of how the specificity of the generated calcium signature can govern the fate of the individual cell and provide a coordinating signal to integrate a population-level response.

## Endnote

1 When this fraction is 50%, this quantity is referred to as the full-width half-maximum (FWHM). We adopt this terminology but use a slightly higher threshold value of 80% to accommodate the fact that some transients do not reach half their initial maximum.
